# C-reactive Protein for Stroke Detection in the Emergency Department in Patients With Dizziness Without Neurological Deficits

**DOI:** 10.3389/fneur.2021.662510

**Published:** 2021-05-31

**Authors:** Seok-In Hong, June-Sung Kim, Hong Jun Bae, Won Young Kim

**Affiliations:** Department of Emergency Medicine, Asan Medical Center, University of Ulsan College of Medicine, Seoul, South Korea

**Keywords:** C-reactive protein, dizzines, emergency department, neurologic deficit, stroke

## Abstract

**Background:** Stroke diagnosis can be challenging in patients with dizziness without neurologic deficits. The aim of this study was to evaluate the predictive value of C-reactive protein (CRP) for identifying acute stroke in such patients.

**Methods:** Data from adult patients (>18 years) admitted to the emergency department from August 2019 to February 2020 were evaluated. The study subjects were 1,188 patients presenting with dizziness without neurological deficits whose serum CRP level was measured within 2 h of arriving at the emergency department and who underwent brain magnetic resonance imaging. The relationship between CRP and acute stroke was analyzed using univariable and multivariable models.

**Results:** Acute stroke was detected in 53 (4.4%) patients (40 with brain infarction, 10 with vertebrobasilar insufficiency, 2 with intracerebral hemorrhage, and 1 with subarachnoid hemorrhage). The CRP levels did not differ significantly between the acute stroke and non-stroke groups [0.10 (0.10–0.31) vs. 0.10 (0.10–0.16), *P* = 0.074]. The area under receiver operating characteristic curve of CRP for acute stroke was not statistically significant (0.567, *P* = 0.101). On multivariable analysis, the following variables were associated with acute stroke: age (odds ratio [OR], 1.041; 95% confidence interval [CI], 1.011–1.071), history of cerebrovascular accidents (OR, 1.823; 95% CI, 1.068–3.110), white blood cell count (OR, 1.126; 95% CI, 1.017–1.248), and hemoglobin (OR, 1.316; 95% CI, 1.056–1.640). However, CRP (*P* = 0.183) was not associated with acute stroke.

**Conclusion:** Serum CRP levels do not have significant discriminative value for identifying acute stroke in patients with dizziness without definite neurologic deficits.

## Introduction

Acute stroke is the world's leading cause of death and disability (1). Early diagnosis warrants immediate initiation of reperfusion therapy to decrease the risk of mortality (2). Dizziness is responsible for ~5% of unselected presentations to emergency departments (EDs) and outpatient clinics (3). Only 0.7–3.2% of ED cases with dizziness are related to stroke (4). However, misdiagnosis may have a serious impact on treatment, disease outcome, and patient quality of life due to the dangerous consequences of posterior circulation strokes and basilar occlusions (5, 6).

Stroke diagnosis can be challenging, especially in a patient with dizziness without definitive neurologic deficits. Magnetic resonance imaging (MRI) is helpful to exclude a central origin of dizziness in such patients. Given the large number of patients with dizziness presenting to the ED, obtaining brain images in patients with low suspicion of stroke can be costly. Moreover, imaging of such patients consumes valuable time and resources in crowded EDs. Although some criteria and bedside physical examinations have been developed to rule out stroke in patients with dizziness (7, 8), determining which patients require further investigation is still difficult.

Serum biomarkers are often used as valuable adjuncts to routine clinical examination and imaging tests. C-reactive protein (CRP), an acute-phase reactant, plays an important role in various diseases (9). Elevated CRP is associated with the development of inflammation, atherosclerosis, and relevant disorders (10). CRP can predict the risk of atherosclerotic cardiovascular disease (ASCVD). The Centers for Disease Control has proposed the use of CRP to stratify the risk of ASCVD (11). From the standpoint of neuroinflammation, inflammatory markers would be expected to be associated with stroke (12, 13), suggesting that CRP could be used as a prognostic marker in acute stroke. A meta-analysis reported an independent association of baseline CRP level with an elevated risk of ischemic stroke (14), and a systematic review that investigated the relationship between multiple biomarkers and the risk of early neurologic deterioration following acute stroke reported that CRP was significantly associated with the risk (15). A high serum CRP level has been associated with the risk of developing a stroke. Jimenez et al. reported that a baseline serum high-sensitivity CRP (hsCRP) level >3.0 mg/L is associated with the development of incident stroke (16), and Liu et al. showed that an hsCRP level >1.0 mg/L is associated with an elevated risk of all strokes (17). However, the predictive value of CRP for identifying acute stroke is limited (18).

Acute stroke cannot be completely excluded without imaging tests; thus, identifying patients who need additional brain imaging using a specific biomarker would be helpful. As mentioned above, a high CRP level is associated with an increased risk of developing stroke; nevertheless, whether the CRP level at the time of ED presentation is useful for stroke detection remains unknown. Associations between CRP and the risk of developing a certain stroke subtype have already been investigated, and elevated CRP levels have been reported in small-vessel disease or posterior circulation infarctions (19). We assumed that stroke of the posterior circulation or a specific subtype could result in dizziness-related symptoms; thus, CRP would be elevated in patients with dizziness without definite neurological deficits. In this study, we evaluated the predictive value of CRP for identifying acute stroke in patients with dizziness, particularly those without neurological deficits.

## Materials and Methods

### Study Settings and Patient Population

In this single-center, retrospective cohort study, we reviewed adult patients (>18 years old) with dizziness or related symptoms (e.g., vertigo, lightheadedness, disequilibrium, or gait instability) as a chief complaint who presented to the ED from August 2019 to February 2020. The exclusion criteria were as follows: patients with definitive neurologic deficits, those who had no MRI data or CRP measurements, and those who had a concomitant infectious disease.

Neurologic deficits were defined as impairments of the central nervous system, such as motor/sensory change of extremities, dysarthria, facial droop, gait disturbances, and impaired consciousness (Glasgow Coma Scale <13). An emergency medicine physician or a neurologist on duty routinely performed neurologic examinations for all patients with dizziness symptoms and described the presence of neurologic deficits in the medical records. Concomitant infections were identified by reviewing medical records for records of infectious symptoms, signs, or test results (e.g., fever with upper respiratory symptoms, laboratory results supporting ongoing infection, or documented infection through imaging tests). CRP is one of the universal markers for inflammation. An infection typically involves inflammation, which often leads to a profound increase in CRP. We attempted to determine whether an increase in the CRP level was a result of acute stroke; thus, patients with concomitant infections were excluded to eliminate a possible covariate.

The Ethics Committee of Asan Medical Center approved the study protocols and waived the need for informed consent. The personal information of all patients was anonymized and removed prior to analysis.

### Data Collection

We collected demographic data (age and sex), the time between the onset of dizziness and the ED visit, and past medical history (hypertension, diabetes, hyperlipidemia, atrial fibrillation, coronary artery disease, cerebrovascular accident, chronic kidney disease, liver cirrhosis, and malignancy) from the hospital records.

Laboratory results [white blood cell (WBC) count, hemoglobin, platelet, glucose, blood urea nitrogen, creatinine, albumin, sodium, potassium, and chloride] including serum CRP levels were collected from the hospital records. All laboratory tests were routinely performed when an MRI test was necessary, within 2 h of a patient's arrival at the ED. CRP was assessed using an immunoturbidimetric assay (AU 5800; Beckman Coulter Inc., Brea, CA, USA) at the certified clinical laboratory at Asan Medical Center. The reported range for the CRP level was 0.1–70.0 mg/dL.

We reviewed MRI images stored in the hospital picture archiving and communication system; these images were interpreted by affiliated radiologists at the time the MRIs were conducted. At our institution, a brain MRI scan is considered a routine imaging workup for patients with dizziness. In Korea, the cost of MRI is covered by the National Health Insurance Service if a patient has a certain symptom covered by the insurance. Therefore, patients are relatively easily examined with laboratory or imaging tests, except if they decline an examination or have a contraindication for MRI.

Acute stroke was defined as a cerebral/cerebellar infarction, vertebrobasilar insufficiency (VBI), or brain hemorrhage documented by brain MRI. We regarded VBI as acute stroke because it appears likely to result in repetitive ischemic symptoms and infarction. Additionally, VBI has also been considered as a posterior circulation transient ischemic attack over decades (20). Acute stroke was confirmed by a neurologist on duty as the etiology of the patient's symptoms and MRI findings.

### Statistical Analysis

A population-based study reported that stroke was diagnosed in 3.2% of patients with dizziness in ED (6). Considering the central limit theorem, at least 30 cases were required to reach the minimal statistical power, with the total number of patients estimated to be ~900 (21). Therefore, we selected the study period to meet the estimated number.

Data are presented as numbers with percentages for categorical variables and medians with interquartile ranges for continuous variables. Statistical differences between the group of patients with acute stroke and those without were analyzed using Fisher's exact test (categorical variables) and the Mann–Whitney *U*-test (continuous variables) as appropriate. The median serum CRP level was compared between the acute stroke and non-stroke groups using Mann–Whitney *U*-test. The relationship between the serum CRP level and acute stroke was also examined using univariable and multivariable logistic regression analyses, and the results are described as odds ratios (ORs) and 95% confidence intervals (CIs). Variables with *P* < 0.1 in the univariable analyses were selected for multivariable analysis. Multivariable logistic analyses were conducted with continuous and categorical variables by using optimal cutoffs. Using receiver operating characteristic (ROC) curves, the area under the ROC curves was determined. The optimal cutoff values of CRP and other variables selected for the multivariable analysis were identified using Youden's index. The sensitivity and specificity were calculated via standard statistical methods. Two-tailed *P* < 0.05 were considered statistically significant. As patients with a CRP level of 0.10 mg/dL—the lower limit of the reporting range—accounted for more than half of the total patients, a subgroup analysis was conducted of patients with elevated CRP levels (>0.10 mg/dL), excluding those whose CRP levels were not increased. All statistical analyses were performed using IBM SPSS Statistics for Windows, version 21.0 (IBM Corp., Armonk, NY, USA).

## Results

### Patient Population

**Figure 1:**
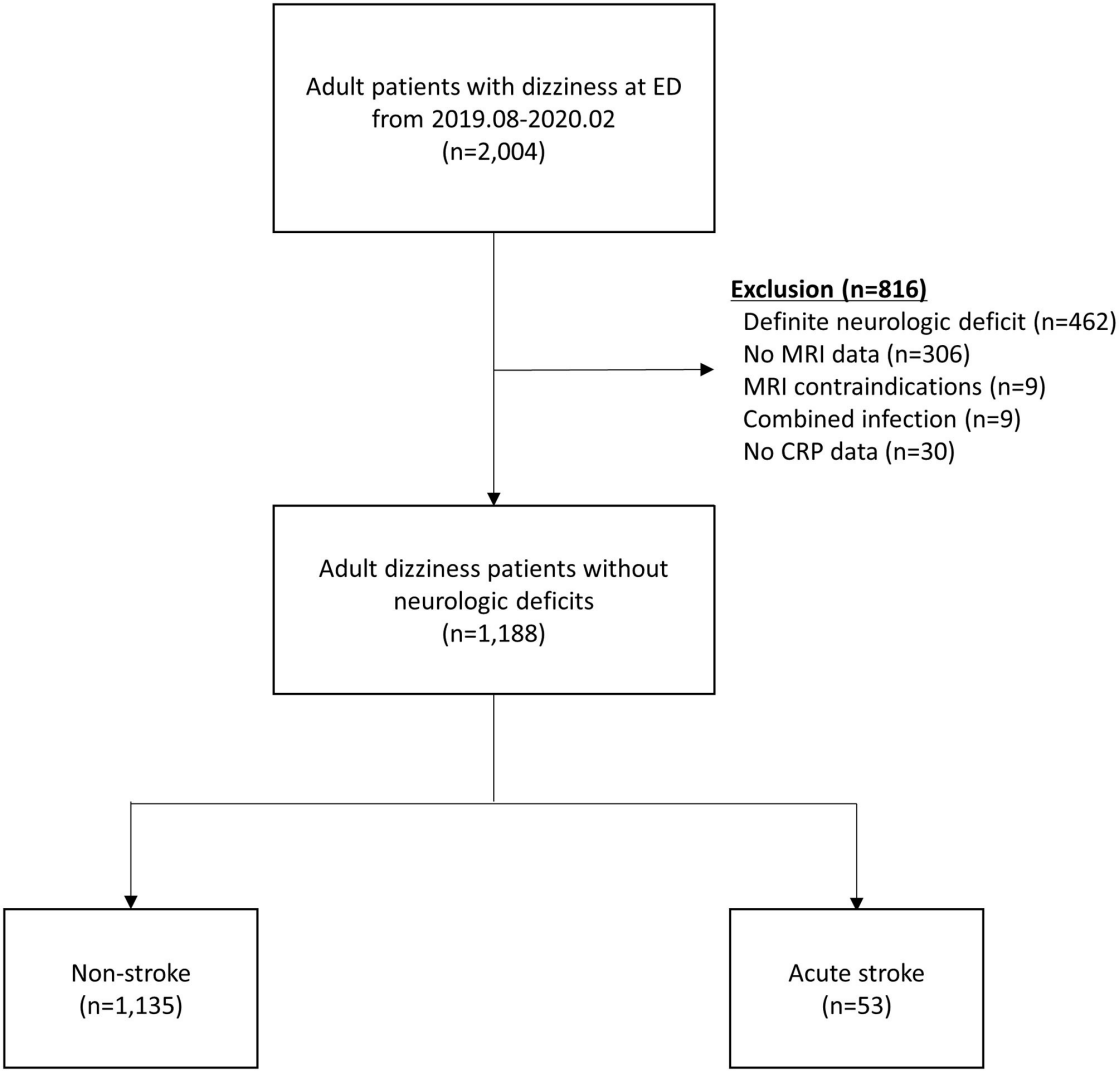
Patient flow diagram. CRP, C-reactive protein; ED, emergency department; MRI, magnetic resonance imaging.

Of 2,004 patients whose chief complaints were dizziness, a total of 1,188 patients without definite neurologic deficits were included in the study. The following patients were excluded: 462 with definite neurologic deficits, 315 without MRI data, 30 without CRP levels measured, and 9 with concomitant infections (Figure 1). Fifty-three (4.4%) patients were diagnosed with acute stroke (40 with brain infarction, 10 with VBI, 2 with intracerebral hemorrhage, and 1 with subarachnoid hemorrhage). Of the 40 patients with a brain infarction, five were diagnosed with an anterior circulation infarction (one with an anterior cerebral artery infarction and four with middle cerebral artery infarctions), six with posterior circulation infarction (three with posterior cerebral artery and three with posterior inferior cerebellar artery infarctions), 12 with a small-vessel infarction, and 10 with multiple/embolic infarctions. In the remaining seven patients with ischemic stroke, the individual arterial vessel could not be identified or classified on imaging. Comparisons of clinical characteristics and laboratory values between the acute stroke and non-stroke groups according to the MRI findings are shown in Table 1. The median age was significantly older in the stroke group than the non-stroke group (71.0 vs. 64.0 years, *P* < 0.002). The median time intervals between symptom onset and the ED visit were not significantly different between the study groups (12.0 vs. 18.0 h, *P* = 0.379). Patients with acute stroke were more likely than patients in the non-stroke group to have hypertension, atrial fibrillation, a history of cerebrovascular accidents, and higher WBC counts and hemoglobin and creatinine levels.

**Table 1:** Baseline clinical characteristics and laboratory findings in the study population.

**Characteristics**	**Total** ** (*n* = 1,188)**	**Non-stroke** ** (*n* = 1,135)**	**Acute stroke** ** (*n* = 53)**	^*^P-value
**Demographics**				
Age (years)	64.0 (56.0–72.0)	64.0 (56.0–72.0)	71.0 (61.0–75.8)	0.002
Male	508 (42.8)	478 (42.1)	30 (56.6)	0.046
Time between onset of dizziness and ED visit (h)	17.0 (5.0–48.0)	18.0 (5.0–48.0)	12.0 (3.0–46.0)	0.379
**Medical history, no. (%)**				
Hypertension	511 (43.0)	481 (42.4)	30 (56.6)	0.047
Diabetes mellitus	185 (15.6)	175 (15.4)	10 (18.9)	0.445
Hyperlipidemia	259 (21.8)	242 (21.3)	17 (32.1)	0.087
Atrial fibrillation	56 (4.7)	49 (4.3)	7 (13.2)	0.010
Coronary artery disease	101 (8.5)	97 (8.6)	4 (7.6)	0.894
Cerebrovascular accident	100 (8.4)	88 (7.8)	12 (22.6)	0.001
Chronic kidney disease	39 (3.3)	38 (3.3)	1 (1.9)	0.559
Liver cirrhosis	29 (2.4)	26 (2.3)	3 (5.7)	0.125
Malignancy	123 (10.4)	115 (10.1)	8 (15.1)	0.247
**Laboratory findings, median (IQR)**				
WBC count ( ×10^3^/μL)	6.8 (5.4–8.4)	6.7 (5.4–8.3)	7.7 (6.0–9.8)	0.003
Hemoglobin (g/dL)	13.5 (12.5–14.5)	13.5 (12.5–14.5)	14.1 (12.3–15.3)	0.046
Platelet ( ×10^3^/μL)	225.0 (190.0–263.0)	226.0 (190.5–263.0)	223.0 (172.8–264.8)	0.683
Glucose (mg/dL)	122.0 (105.0–149.0)	122.0 (105.0–148.0)	131.5 (112.3–158.0)	0.129
BUN (mg/dL)	15.0 (12.0–19.0)	15.0 (12.0–19.0)	16.0 (12.0–19.0)	0.582
Creatinine (mg/dL)	0.80 (0.68–0.97)	0.80 (0.68–0.96)	0.84 (0.75–1.06)	0.007
Albumin (g/dL)	4.00 (3.80–4.20)	4.00 (3.80–4.20)	3.90 (3.75–4.10)	0.277
Sodium (mmol/L)	141.0 (139.0–142.0)	141.0 (139.0–142.0)	140.0 (139.0–142.0)	0.407
Potassium (mmol/L)	4.2 (4.0–4.5)	4.2 (4.0–4.5)	4.2 (3.9–4.4)	0.682
Chloride (mmol/L)	103.0 (102.0–105.0)	103.0 (102.0–105.0)	103.0 (101.0–105.0)	0.411

*Data are presented as n (%) or the median with the interquartile range*.

* *P-values were calculated from the comparison between non-stroke and acute stroke groups*.

*BUN, blood urea nitrogen; ED, emergency department; IQR, interquartile range; WBC, white blood cell*.

### Outcomes

Serum CRP levels were compared between the two groups: non-stroke and acute stroke. The median CRP values were the same in the two groups at 0.10 mg/dL; no significant difference was detected between the two groups (*P* = 0.074). Moreover, 64.2% of the patients in the study population had a CRP level of 0.10 mg/dL. The percentages of patients who had a CRP level >0.10 mg/dL were 35.4% in the non-stroke group and 44.2% in the stroke group. A subgroup analysis was conducted for the patients whose CRP level was above 0.10 mg/dL, and a significant difference was found between these two groups (*P* = 0.019). The proportion of males was also presented (22) and was not significantly different between the non-stroke and stroke groups of patients with a CRP level >0.10 mg/dL (*P* = 0.396; Table 2).

**Table 2:** C-reactive protein levels in the study population stratified by stroke status.

	**Total or subtotal**	**Non-stroke**	**Acute stroke**	^*^P
**All patients regardless of CRP level**				
Number of patients	1,188	1,135	53	
Male, n (%)	508 (42.8)	478 (42.1)	30 (56.6)	0.046
CRP, median (IQR)	0.10 (0.10–0.16)	0.10 (0.10–0.16)	0.10 (0.10–0.31)	0.074
**Patients with CRP** **>0.10 mg/dL**				
Number of patients (%)	425 (35.8)	402 (35.4)	23 (44.2)	
Male, *n* (%)	201 (47.3)	188 (46.8)	13 (56.5)	0.396
CRP, median (IQR)	0.22 (0.15–0.43)	0.21 (0.15–0.42)	0.31 (0.23–1.13)	0.019

*The numbers of patients and males and the median level of CRP levels in the study population are presented. Below the row labeled CRP >0.10 mg/dL, the number and median CRP levels of only patients whose CRP levels were above 0.10 mg/dL are presented*.

* *P-values were calculated from the comparison between non-stroke and acute stroke groups*.

*CRP, C-reactive protein; IQR, interquartile range *.

In the multivariable analysis, age (OR, 1.041; 95% CI, 1.011–1.071, *P* = 0.006), history of cerebrovascular accidents (OR, 1.823; 95% CI, 1.068–3.110, *P* = 0.028), WBC count (OR, 1.126; 95% CI, 1.017–1.248, *P* = 0.023), and hemoglobin level (OR, 1.316; 95% CI, 1.056–1.640, *P* = 0.015) were associated with acute stroke, but CRP level (*P* = 0.183) was not (Table 3). The area under the ROC curve of CRP for acute stroke was not statistically significant (0.567, *P* = 0.101). We categorized continuous variables using the optimal cutoff values derived from the ROC curves and performed backward stepwise multivariable logistic analysis. The following variables were significantly associated with acute stroke: age (cutoff, 65.0; OR, 2.361; 95% CI, 1.261–4.419, *P* = 0.007), history of cerebrovascular accidents (OR, 2.618; 95% CI, 1.261–5.434, *P* = 0.010), and WBC count (OR, 2.207; 95% CI, 1.228–3.996, *P* = 0.008), whereas CRP was not (Table 3). The cutoffs used in the logistic regression analysis and performance parameters of the variables are shown in Table 4. Although a CRP level >0.105 mg/dL had the highest specificity (64.58%) relative to those of other laboratory results, it had a poor sensitivity (44.23%) and positive predictive value (1.25%).

**Table 3:** Multivariable logistic analysis for predicting acute stroke.

**Variables**	**Multivariable**			^*^Multivariable ** (Categorized using cutoffs)**		
	**Adjusted** ** OR**	**95% CI**	***P***	**Adjusted** ** OR**	**95% CI**	***P***
Age	1.041	1.011–1.071	0.006	2.361	1.261–4.419	0.007
Male	1.134	0.594–2.165	0.704			
Hypertension	1.004	0.530–1.901	0.991			
Hyperlipidemia	1.440	0.758–2.738	0.265			
Atrial fibrillation	2.081	0.843–5.138	0.112	2.285	0.941–5.551	0.068
CVA	1.823	1.068–3.110	0.028	2.618	1.261–5.434	0.010
WBC count	1.126	1.017–1.248	0.023	2.207	1.228–3.996	0.008
Hemoglobin	1.316	1.056–1.640	0.015			
Creatinine	1.009	0.995–1.024	0.218			
CRP	1.314	0.879–1.966	0.183			

* *The third column of the table shows the multivariable analysis for categorized variables using certain cutoffs calculated from the receiver operating characteristic curve. A cutoff for each variable is presented in Table 4. CI, confidence interval; CRP, C-reactive protein; CVA, cerebrovascular accident; OR, odds ratio; WBC, white blood cell*.

**Table 4:** Performance parameters of predictors of acute stroke in the study population.

**Variables**	**Sensitivity (%)**	**Specificity (%)**	**PPV (%)**	**NPV (%)**	**PLR**	**NLR**
WBC >6.95	63.46	53.99	1.38	0.68	5.92	97.00
Hb >13.65	58.77	54.43	1.22	0.81	5.29	96.42
Cr >0.825	58.49	54.96	1.30	0.76	5.70	96.60
CRP >0.105	44.23	64.58	1.25	0.86	5.41	96.19

*Hb, hemoglobin; Cr, creatinine; CRP, C-reactive protein; NLR, negative likelihood ratio; NPV, negative predictive value; PLR, positive likelihood ratio; PPV, positive predictive value; WBC, white blood cell*.

Supplementary Table 1 shows the CRP levels according to the affected vascular territories on MRI images. The median CRP levels were 0.18, 0.51, and 0.18 mg/dL in patients with middle cerebral artery infarctions, posterior cerebral artery infarctions, and embolic infarctions, respectively. The median CRP level in patients with stroke affecting other vascular territories was 0.10 mg/dL.

## Discussion

We sought to evaluate the usefulness of CRP as a tool for predicting acute stroke in patients without neurologic deficits. The CRP level did not significantly differ between the stroke group and the non-stroke group. Multivariable analysis also showed that CRP was not significantly associated with acute stroke in patients with dizziness without neurological deficits.

Cerebral stroke triggers an inflammatory response characterized by activation and release of acute phase proteins such as CRP (23). Increased CRP levels are found in as many as three-quarters of patients with acute stroke; thus, increases in CRP may reflect a systemic inflammatory response following stroke, the extent of tissue injury, or a pre-existing degree of atherosclerosis (24). Previous studies have indicated that elevated CRP levels are correlated with stroke severity and independently predict mortality and the recurrence of stroke (25, 26). Identifying effective biomarkers for the early suspicion of acute stroke is clinically important; however, the role of CRP in stroke detection remains unknown owing to the lack of reports, especially those involving patients with dizziness. In a recent single-center observational study that investigated the relationship among various laboratory results and stroke, the authors found no difference in CRP levels between patients with stroke and those without stroke with acute vestibular syndrome [26 vs. 29%, *p* = 0.726; (27)]. This is consistent with our study results that the CRP level did not differ between the acute stroke and non-stroke groups. In contrast, hsCRP was suggested to be a useful indicator of acute stroke in a retrospective study, and a CRP level of 0.18 mg/dL was presented as a cutoff (28). The lower limit of reported CRP levels differed between the two studies; the former reported the values down to 0.50 mg/dL, and the latter reported down to 0.01 mg/dL. These studies included only patients with acute vestibular syndrome; in contrast, our study included all patients with dizziness-related symptoms. Conducting MRI only in patients whose CRP levels are above a certain threshold would be clinically useful. Our clinical laboratory has been reporting CRP values below 0.50 mg/dL; thus, we expected to find a positive relationship between CRP and acute stroke. However, our study did not reveal that CRP is a valid predictive tool.

We considered several possible explanations for these statistically insignificant findings. First, in contrast to other studies that included all patients with stroke regardless of neurologic deficits (16, 17), we included only patients with dizziness without neurological deficits. Based on studies reporting that increased CRP levels might reflect the severity of the stroke (29, 30), 38 (77%) of the patients with stroke included in this study were diagnosed with cerebellar, embolic, or small-vessel infarctions; these relatively small infarctions may have resulted in only a slight increase in CRP levels (31). Although the objective stroke severity of the included patients at the time of ED presentation was not calculated, most of the included patients with stroke were considered to have non-severe stroke due to the absence of neurologic deficits. Stroke severity is often measured using the National Institutes of Health Stroke Scale (NIHSS) (32). The anticipated score of these patients would be zero to two, even if having non-specific gait instability was regarded as a neurologic deficit. In addition, VBI was regarded as acute stroke in our study, and this disease may only result in transient ischemic symptoms without an identifiable infarction lesion on brain MRI and may also have contributed only to a minimal increase in CRP levels. A recent review article reported that the main clinical symptoms of VBI were dizziness, vertigo, imbalance, and weakness in the body (33). Therefore, it was necessary to consider VBI as an important disease in the spectrum of acute stroke to fulfill the purpose of the current study. These are possible reasons for this negative result, considering the location of stroke that can cause dizziness without neurologic deficits. This idea is also supported by the fact that >50% of the included patients had a CRP level <0.10 mg/dL.

Second, it was not possible to measure CRP levels <0.10 mg/dL. Our clinical laboratory reports CRP values down to 0.10 mg/dL. More than half (64.2%) of the included patients had a CRP level of 0.10 mg/dL, which might explain our negative results in contrast to those of a previous study reporting increased CRP levels in small-vessel disease or posterior circulation infarctions (19). A high-sensitivity CRP test instead of a routine CRP test was performed in the previous study. The lower sensitivity of CRP than that of hsCRP (analytical sensitivity, 0.01 mg/dL) could explain this discrepancy. Otherwise, for patients with a CRP level >0.10 mg/dL, we observed a significant difference between the acute stroke group and the non-stroke group. Even in this case, logistic regression analysis did not reveal a meaningful relationship between CRP and acute stroke. A high-sensitivity test that can measure CRP values below 0.10 mg/dL could yield a statistically significant difference between the study groups; however, the hsCRP test is not routinely performed in the ED setting. The predictive value of hsCRP in the ED for justifying a recommendation of brain MRI remains unknown, and future research is needed to identify the optimal cutoff of hsCRP.

Our study has several notable features. The first is the timing of the blood tests. In many other studies, the timing of blood sampling varied, usually within several hours to 48 h of admission. The results of blood tests vary depending on the time of sampling because of their nature; therefore, it is critical to maintain a specific sampling time range. In our study, the time intervals between the onset of dizziness and ED visit were not significantly different between the two study groups, and all blood samples were obtained within 2 h of the ED visits. Second, we are the first to investigate the relationship between CRP and acute stroke in isolated patients with dizziness in the ED. Although the CRP level did not significantly predict stroke, these findings will be helpful for future research.

The limitations of the current study are related mainly to its retrospective nature and that the study was conducted in a single institution: it is possible that selection bias may have affected the results, which limits the generalization of our findings to other patient populations. We tried to include patients with a wide range of dizziness-related symptoms; however, some patients with chief complaints other than dizziness (e.g., vomiting and generalized weakness) were not included in our study. For instance, a patient who presented with vomiting as a chief complaint might have had accompanying dizziness. However, the patient was excluded because we extracted the data only according to the chief complaints that met the inclusion criteria rather than according to all symptoms each patient was experiencing. Furthermore, the diagnosis of patients without stroke was not collected. In our study, only the diagnosis of acute stroke was investigated. Due to the variety of final diagnoses in patients with dizziness symptoms who visited the ED, it was impossible to obtain the data for all diagnoses in patients not diagnosed with acute stroke. Moreover, the confirmation of a certain diagnosis was often not made in the ED. The number of patients without stroke was 20 times larger than that of patients with stroke in our study. Considering the large number of patients and the diagnostic variety among patients without stroke, it might have been more persuasive to explain our negative results for CRP by obtaining diagnostic data other than acute stroke. In addition to the requirement for other diagnoses in the study population, the Head Impulse–Nystagmus–Test of Skew (HINTS) might have provided additional information to explain our study results. The HINTS test is known to be useful for differentiating posterior circulation stroke from peripheral vertigo with high sensitivity and specificity (34). However, the HINTS has a limitation that the test is applicable only to specific patients. The patient must present with acute vestibular syndrome and must currently be symptomatic with nystagmus (7). Therefore, the HINTS test was not routinely performed in our study patients due to the inclusion criteria, which attempted to include patients with a wide range of dizziness symptoms encompassing acute vestibular syndrome.

## Conclusions

Among patients with dizziness without neurologic deficits visiting the ED, only 4.4% were diagnosed with acute stroke. Initial CRP levels did not have a significant discriminative value for identifying acute stroke in these patients.

## Data Availability Statement

The raw data supporting the conclusions of this article will be made available by the authors, without undue reservation.

## Ethics Statement

The studies involving human participants were reviewed and approved by the ethics committee of Asan Medical Center. Written informed consent for participation was not required for this study in accordance with the national legislation and the institutional requirements.

## Author Contributions

All authors contributed to study conception and design. Data acquisition was performed by JSK and HJB. Data analysis and interpretation were performed by SIH. The first draft of the manuscript was written by SIH and JSK. WYK revised the manuscript critically for intellectual content. All authors read and approved the final manuscript.

## Conflict of Interest

The authors declare that the research was conducted in the absence of any commercial or financial relationships that could be construed as a potential conflict of interest.
