# Supplementary material for: C-reactive Protein for Stroke Detection in the Emergency Department in Patients With Dizziness Without Neurological Deficits
Source: Front Neurol. 2021 May 31;12:662510. doi: 10.3389/fneur.2021.662510 (PMC8200534; doi:10.3389/fneur.2021.662510)
Supplement: Supplementary file 1 [file Table_1.DOCX]

Table S1. Affected vascular territories on images and CRP levels among patients with ischemic stroke

| **Circulatory classification** | **Vascular territory** | **Number of patients (%)** | **CRP (mg/dL)** |
| --- | --- | --- | --- |
| Anterior circulation | Anterior cerebral artery | 1 (2) | 0.10 (0.10–0.10) |
|  | Middle cerebral artery | 4 (8) | 0.18 (0.10–0.78) |
| Posterior circulation | Posterior cerebral artery | 3 (6) | 0.51 (0.10–1.47) |
|  | Posterior inferior cerebellar artery | 3 (6) | 0.10 (0.10–0.28) |
|  | Vertebrobasilar artery | 10 (20) | 0.10 (0.10–0.12) |
| Not specified | Small vessel disease | 12 (24) | 0.10 (0.10–0.24) |
|  | Embolic | 10 (20) | 0.18 (0.10–0.31) |
|  | Others | 7 (14) | 0.10 (0.10–0.80) |

The number of patients is presented as number with percentage. The level of CRP is presented as median with interquartile range for each vascular territory.

Abbreviations: CRP, C-reactive protein
